# Supplementary figures and images for: Effect on growth of exposure to maternal antiretroviral therapy in breastmilk versus extended infant nevirapine prophylaxis among HIV-exposed perinatally uninfected infants in the PROMISE randomized trial
Source: PLoS One. 2021 Aug 20;16(8):e0255250. doi: 10.1371/journal.pone.0255250 (PMC8378741; doi:10.1371/journal.pone.0255250)

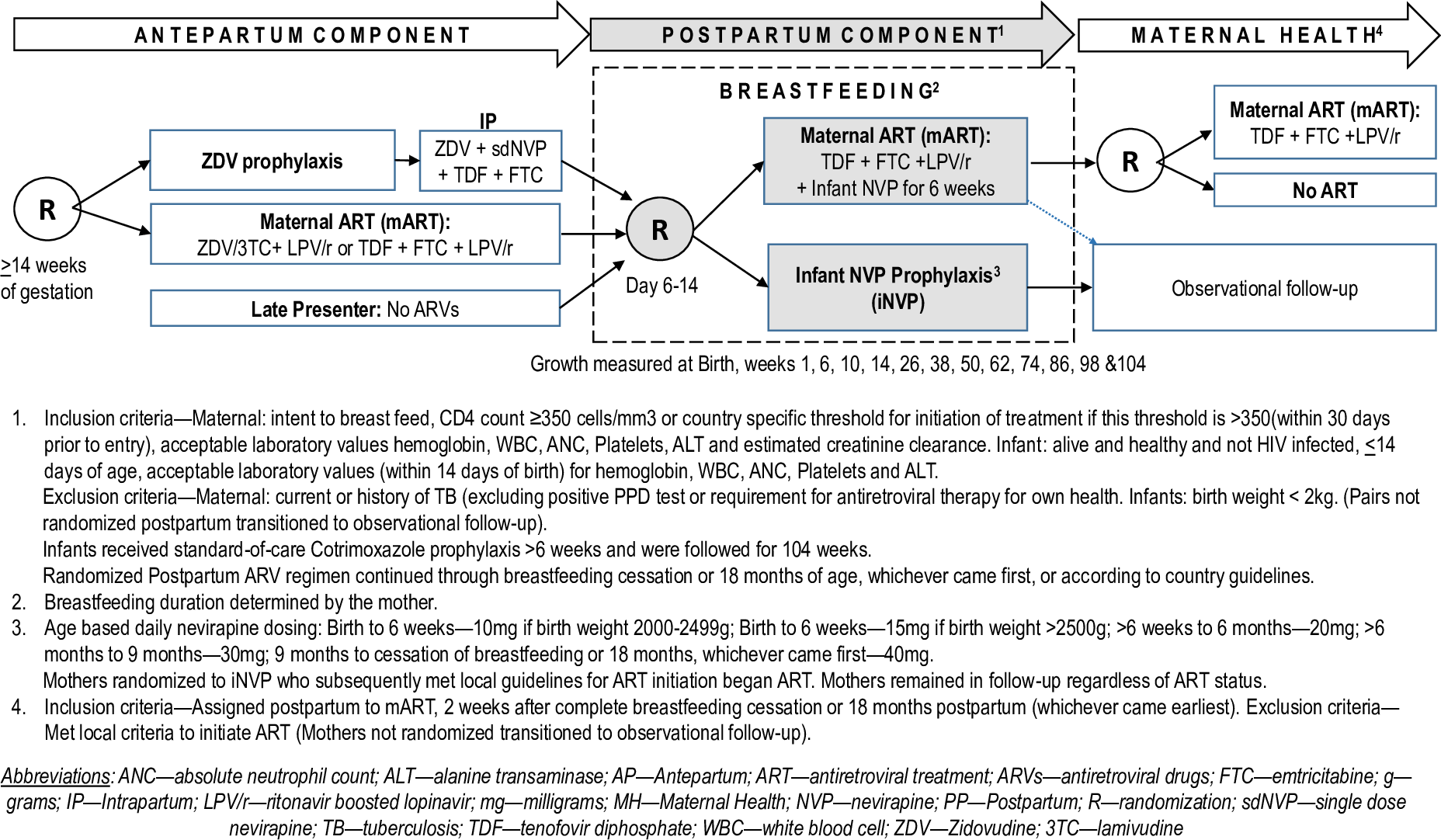

Supplement: S1 Fig — (TIF) [file pone.0255250.s002.tif]
