# Supplementary material for: Effect on growth of exposure to maternal antiretroviral therapy in breastmilk versus extended infant nevirapine prophylaxis among HIV-exposed perinatally uninfected infants in the PROMISE randomized trial
Source: PLoS One. 2021 Aug 20;16(8):e0255250. doi: 10.1371/journal.pone.0255250 (PMC8378741; doi:10.1371/journal.pone.0255250)
Supplement: S1 Table — (DOCX) [file pone.0255250.s003.docx]

**S2 Table. Sensitivity analysis comparisons of infant growth measurements by Postpartum Component randomization**

|  | | **mART** | |  | **iNVP** | |  | |
| --- | --- | --- | --- | --- | --- | --- | --- | --- |
| **Study Week** | **Outcome** | **N** | **n (%)** |  | **N** | **n (%)** |  | **Odds Ratio (95% CI)** |
| Week 10 | LAZ < -1 (Sensitivity) | 1158 | 650 (56.1) |  | 1168 | 635 (54.4) |  | 1.07 (0.91, 1.26) |
|  | LAZ < -2 | 1158 | 322 (27.8) |  | 1168 | 317 (27.1) |  | 1.03 (0.86, 1.24) |
|  | LAZ < -3 (Sensitivity) | 1158 | 122 (10.5) |  | 1168 | 119 (10.2) |  | 1.04 (0.80, 1.36) |
| Week 26 | LAZ < -1 (Sensitivity) | 1150 | 555 (48.3) |  | 1162 | 550 (47.3) |  | 1.04 (0.88, 1.22) |
|  | LAZ < -2 | 1150 | 258 (22.4) |  | 1162 | 214 (18.4) |  | 1.28 (1.05, 1.57) |
|  | LAZ < -3 (Sensitivity) | 1150 | 76 (6.6) |  | 1162 | 65 (5.6) |  | 1.19 (0.85, 1.68) |
| Week 74 | LAZ < -1 (Sensitivity) | 864 | 583 (67.5) |  | 879 | 582 (66.2) |  | 1.06 (0.87, 1.29) |
|  | LAZ < -2 | 864 | 305 (35.3) |  | 879 | 281 (32.0) |  | 1.16 (0.95, 1.42) |
|  | LAZ < -3 (Sensitivity) | 864 | 99 (11.5) |  | 879 | 90 (10.2) |  | 1.13 (0.84, 1.53) |
| Week 104 | LAZ < -1 (Sensitivity) | 655 | 459 (70.1) |  | 653 | 440 (67.4) |  | 1.13 (0.90, 1.43) |
|  | LAZ < -2 | 655 | 228 (34.8) |  | 653 | 215 (32.9) |  | 1.09 (0.87, 1.37) |
|  | LAZ < -3 (Sensitivity) | 655 | 83 (12.7) |  | 653 | 61 (9.3) |  | 1.41 (0.99, 2.00) |

|  | | **mART** | |  | **iNVP** | |  | |
| --- | --- | --- | --- | --- | --- | --- | --- | --- |
| **Study Week** | **Outcome** | **N** | **n (%)** |  | **N** | **n (%)** |  | **Odds Ratio (95% CI)** |
| Week 10 | WAZ < -1 (Sensitivity) | 1157 | 291 (25.2) |  | 1168 | 282 (24.1) |  | 1.06 (0.87, 1.27) |
|  | WAZ < -2 | 1157 | 93 (8.0) |  | 1168 | 67 (5.7) |  | 1.44 (1.04, 1.99) |
|  | WAZ < -2.5 (Sensitivity) | 1157 | 41 (3.5) |  | 1168 | 31 (2.7) |  | 1.35 (0.84, 2.16) |
| Week 26 | WAZ < -1 (Sensitivity) | 1151 | 302 (26.2) |  | 1161 | 309 (26.6) |  | 0.98 (0.82, 1.18) |
|  | WAZ < -2 | 1151 | 81 (7.0) |  | 1161 | 63 (5.4) |  | 1.32 (0.94, 1.85) |
|  | WAZ < -2.5 (Sensitivity) | 1151 | 39 (3.4) |  | 1161 | 28 (2.4) |  | 1.42 (0.87, 2.32) |
| Week 74 | WAZ < -1 (Sensitivity) | 864 | 291 (33.7) |  | 878 | 274 (31.2) |  | 1.12 (0.92, 1.37) |
|  | WAZ < -2 | 864 | 79 (9.1) |  | 878 | 50 (5.7) |  | 1.67 (1.15, 2.41) |
|  | WAZ < -2.5 (Sensitivity) | 864 | 37 (4.3) |  | 878 | 21 (2.4) |  | 1.83 (1.06, 3.15) |
| Week 104 | WAZ < -1 (Sensitivity) | 654 | 213 (32.6) |  | 653 | 205 (31.4) |  | 1.06 (0.84, 1.33) |
|  | WAZ < -2 | 654 | 54 (8.3) |  | 653 | 31 (4.7) |  | 1.81 (1.14, 2.85) |
|  | WAZ < -2.5 (Sensitivity) | 654 | 19 (2.9) |  | 653 | 15 (2.3) |  | 1.27 (0.64, 2.53) |

*Abbreviations: iNVP—infant nevirapine prophylaxis; LAZ—length-for-age z-score; mART—maternal antiretroviral treatment; N—number; WAZ—weight-for-age z-score; %—percent; 95% CI—95% confidence interval*
