# Supplementary material for: Effect on growth of exposure to maternal antiretroviral therapy in breastmilk versus extended infant nevirapine prophylaxis among HIV-exposed perinatally uninfected infants in the PROMISE randomized trial
Source: PLoS One. 2021 Aug 20;16(8):e0255250. doi: 10.1371/journal.pone.0255250 (PMC8378741; doi:10.1371/journal.pone.0255250)
Supplement: Attachment — Submitted filename: Response to Reviewers_PONE-D-21-06961 [R1].pdf [file pone.0255250.s005.pdf]

University of Zimbabwe - Clinical Trials Research Centre
Saving Lives Through Innovative Research Strategies

University of Zimbabwe
Clinical Trials Research Centre

Administrative Office
15 Phillips Avenue, Belgravia, Harare, Zimbabwe
Tel: +263-24-2704890/2704920, Fax: +263-24-2704897

Research Office
10 Routledge Street, Milton Park, Harare, Zimbabwe
Tel: +263-24-252299/27911374, Fax: +263-24-2704897

KNOWLEDGE • DILIGENCE • INTEGRITY
University of Zimbabwe,
Faculty of Medicine and Health Sciences

10 May 2021

PONE-D-21-06961

The Editor
PLOS ONE

Dear Editor,

We thank the two reviewers for their insightful comments each of which we present verbatim in *italicised* font, followed by our response. For ease of referencing, the version of the revised manuscript with tracked changes has line numbers which are mentioned in the responses below.

Yours Sincerely,

Dr Lynda Stranix-Chibanda
MBChB, MMeD Paeds
University of Zimbabwe
Clinical Trials Research Centre
lstranix@uz-ctrc.org

REVIEWER #1:

R1.1 - *Abstract - report the exact p-value instead of p-value≥0.16 in line 65 and p-value≥0.09 in line 67 and anywhere else in the manuscript, unless p-value is less than 0.001 or greater than 0.999.*

Thank you for the comment. We have revised the Abstract [see lines 66 and 68] to show that the several p-values presented there were all >=0.16 and >=0.09, respectively, and trust that this adequately addresses the concern raised.

R1.2 - *Statistical methods - t-tests to compare z-scores doesn't seem appropriate here; why not conduct an analysis of covariance (ANCOVA) by fitting a linear regression model of the post-randomisation z-scores adjusting for baseline/pre-randomisation z-scores and with treatment group-time interaction to estimate how the z-scores differ between groups at the various post-intervention times when they were measured. A likelihood ratio p-value for the treatment-time interaction would assess evidence for whether the treatments differ at the various time points - this would be a statistically more efficient way to analyse these data.*

We understand the approach suggested by Reviewer 1, however, the protocol-specified primary objective was to conduct a cross sectional comparison at postpartum Week 26, with the primary outcome measure of LAZ. Other timepoints and measures were secondary. After discussion by the writing team, we decided to clarify the statistical analysis approach in the revised text [see Statistical Methods lines 221-225 and Abstract line 57] and believe that the explanation is now clear.

R1.3 - *Results - rather than presenting means and confidence intervals, I think table 2 should present means and SEs for each treatment group, followed by the difference, confidence intervals and p-values, for each outcome and time.*

Table 2 has been adjusted in line with Reviewer 1's comment and week 0 infant anthropometric measures were moved to Table 1 [see lines 300-309 in the revised manuscript]. Please note that we took the opportunity to correct an erroneous percentage for stunting in Table 2. The text preceding the table, table title, and footnote were updated accordingly. Table 1 was updated thus: addition of week 0 LAZ, WAZ, and HCAZ under infant characteristics (including % z score < -2); removal of rows for "N" for maternal characteristics (since they are redundant given the column headers and # missing); addition of 10%-90% for infant characteristics (to be consistent with the descriptive statistics presented for mothers); footnote updated accordingly [see lines 271-279].

R1.4 - *Results - actual p-values should be reported all through unless less than 0.001 or greater than 0.999; for example for p≥0.14 in line 323 and p≤0.03 in line 329*

We have attempted to address this concern by clarifying that we present several p-values in the examples mentioned where no actual p-value is shown. Please see the inserted text in the revised manuscript [for example in line 333].

REVIEWER #2:

R2.0 - *The article is generally well written with impressive design and study size. The article is generally well analyzed and presented. Except some few sentences I generally agree with the interpretations and summaries.*

Thank you for the comment.

R2.1 - *The conclusion in the abstract is emphasizing stunting and underweight "differences". Based on the data you present, there seems to be no difference at week 0, week 10, week 74 and week 104 (and no clinically relevant difference at week 26). I highly doubt the 0.9% "difference" at 26 weeks which is not seen earlier or later is causally related to the intervention/drugs. Rather, I find it more probable to assume that this is due to either slight measurement inaccuracies or stochastic effects in timing of growth (as growth velocity is not constant). Thus, I suggest the conclusion in the abstract is modified, e.g. as following:*
"In HEUs, growth effects from postnatal exposure to mART compared to iNVP were comparable for measures on length, weight and head-circumference with no clinically relevant differences between the groups. "

Thank you for the insight and the suggested revision which was considered by the writing team. We opted to use the sentence you provided for the Conclusion in the Abstract [see lines 71-82 in the revised manuscript].

R2.2A - *Similarly, with so comparable mean WAZ-measures between the groups, I am also skeptical to*

the interpretation of difference for underweight. This could be e.g. a threshold effect. If you check two additional thresholds (e.g. -1 and -3) and find similar differences, I would be a little less skeptical to the validity of these differences.

We hope the adjusted interpretation presented in the revised manuscript likely addresses this concern. To explain the -2 SD threshold selection, that is the standard clinical definition for stunting and underweight based on World Health Organisation definitions and is not driven by study data. Thus, we made minor clarifications to the Methods and inserted new reference #21 to illustrate the basis for that threshold in the revised manuscript [see lines 237—239]. However, we did carry out a sensitivity analysis around -2, with thresholds of -1.9 and -2.1 to address potential random noise right around this measurement that could result in disproportionate assignments to one category. Results were very similar, and we determined there was no need to include them in the manuscript.

R2.2B *- As there are multiple comparisons and the study have considerable power and size, you could consider using a p<0.01 significance threshold.*

Indeed, this study does have considerable size. The 0.05 significance level was pre-specified in our analysis plan.

R2.2C *- More importantly, I would recommend that you emphasize clinically relevant differences rather than statistically significant differences. Thus, the subsequent sentences in the conclusion of the abstract should be aligned with the manuscript, and I would slightly modified the results section accordingly.*

We agree with Reviewer 2's helpful guidance. Accordingly, we revised the Conclusions in the Abstract [see lines 71-82] and removed the term 'significant' from several sentences in the Results section [see lines 314-346] to remove any misrepresentation of clinical relevance. We do include p-values in Table 2, as suggested by Reviewer 1 (comment R1.3), but they are not emphasized heavily in the text.

R2.2D *- I think the first sentence of the discussion and the conclusion in the main manuscript is more balanced.*
Except these essential changes, I find the article excellent and well worth publishing.

Thank you for the insightful comments and suggestions which we have taken on board and used to strengthen the manuscript we now resubmit.
