# Supplementary material for: Effect on growth of exposure to maternal antiretroviral therapy in breastmilk versus extended infant nevirapine prophylaxis among HIV-exposed perinatally uninfected infants in the PROMISE randomized trial
Source: PLoS One. 2021 Aug 20;16(8):e0255250. doi: 10.1371/journal.pone.0255250 (PMC8378741; doi:10.1371/journal.pone.0255250)
Supplement: Attachment — Submitted filename: Response to Reviewers [R2].pdf [file pone.0255250.s006.pdf]

University of Zimbabwe - Clinical Trials Research Centre
Saving Lives Through Innovative Research Strategies

University of Zimbabwe
Clinical Trials Research Centre

Administrative Office
15 Phillips Avenue, Belgravia, Harare, Zimbabwe
Tel: +263-24-2704890/2704920, Fax: +263-24-2704897

Research Office
10 Routledge Street, Milton Park, Harare, Zimbabwe
Tel: +263-24-252299/27911374, Fax: +263-24-2704897

KNOWLEDGE · DILIGENCE · INTEGRITY
University of Zimbabwe,
Faculty of Medicine and Health Sciences

30 June 2021

PONE-D-21-06961

The Editor
PLOS ONE

Dear Editor,

Once again, we thank the two reviewers for their additional comments on the re-submitted manuscript [Revision 1], each of which we present verbatim in *italicised* font, followed by our response. For ease of referencing, the version of the revised manuscript [Revision 2] with tracked changes has line numbers which are mentioned in the responses below.

Yours Sincerely,

Dr Lynda Stranix-Chibanda
MBChB, MMed Paeds
University of Zimbabwe
Clinical Trials Research Centre
lstranix@uz-ctrc.org

REVIEWER #1:

[Revision 1] R1.1 - *Please correct the typographical error at the top of page 11 of 24 - you mean Kruskal-Wallis, not Kruskal-Wallace.*

Thank you for identifying this error which is corrected in line 228 of the marked-up revised manuscript [Revision 2].

REVIEWER #2:

[Revision 1] R2.0 - *I think the article has improved and that the first response is fine.*

Noted.

[Revision 1] R2.1 - *I do not fully agree with the approaches on question 2.2, but that being said the discussion of that finding and conclusion still look generally fine. I am aware that z-score -2 is a commonly used threshold for stunting and underweight, but e.g. -3 is also a commonly used threshold for severe underweight/stunting. See also the article by Briend et al. on arguments for not only focusing on a single threshold (Briend A, Van den Broeck J, Fadnes LT: Target weight gain for moderately wasted children during supplementation interventions - a population-based approach. Public health nutrition 2011:1-7.) I do not see the option of adding sensitivity analysis for z-score thresholds of -1.9 and -2.1*

University of Zimbabwe
Clinical Trials Research Centre

UZ-CTRC
University of Zimbabwe - Clinical Trials Research Centre
Saving Lives Through Innovative Research Strategies

Administrative Office
15 Phillips Avenue, Belgravia, Harare, Zimbabwe
Tel: +263-24-2704890/2704920, Fax: +263-24-2704897

Research Office
10 Routledge Street, Milton Park, Harare, Zimbabwe
Tel: +263-24-252299/27911374, Fax: +263-24-2704897

KNOWLEDGE · DILIGENCE · INTEGRITY
University of Zimbabwe,
Faculty of Medicine and Health Sciences

(which is very close to the original analysis) as good alternatives to what I suggested of a sensitivity analyses with z-score thresholds of -3 and -1. Unless you see similar results with thresholds of -3 and -1, I think the presentation of differences in the results section is not optimal. However, if you show similar trends for sensitivity analyses, you will probably convince me of your presentation/interpretation. For the results section as I consider the confidence intervals for the dichotomous outcomes are relatively wide (stunting/overweight), I suggest sensitivity analysis with thresholds further away from the original analysis (and that these preferable are added as a supplementary table) to guide whether these are threshold effects. If so, that could be mentioned shortly in the results/discussion section.

The writing team accepted the reviewer's comment and are grateful for the reference provided. We agree with not focusing on a single threshold for this secondary outcome measure. We conducted additional sensitivity analyses using thresholds further away from the original z-score threshold (-2.0). For length-for-age (LAZ), the thresholds used were -1.0 and -3.0, as suggested. However, the number of infants with weight-for-age (WAZ) below -3.0 was very low (<2%) at all time points, therefore the thresholds used for WAZ were -1.0 and -2.5. The direction of the odds ratio estimate remains consistent across the different thresholds for both LAZ and WAZ. The results of this sensitivity analysis are now mentioned in line 333 of the marked-up revised manuscript [Revision 2]. The sensitivity analysis results are uploaded with this rebuttal letter for review. We have included a table as new Supporting Information for the revised manuscript (caption inserted at the end of the manuscript document), and include figures below illustrating additional thresholds for the LAZ and WAZ comparisons (Figure A and Figure B).

[Revision 1] R2.2 - *Also a note relating to the p-value threshold, even though p<0.05 was predefined, it is still possible to guide interpretation not only dichotomously but to discuss degree of association (and chance of random associations versus causal inferences).*

That being said, the study and article is generally very good and well worth publishing. I just think adding a sensitivity analysis which is more different from the original analysis would lift the paper even more.

We reviewed the manuscript with this note in mind. In Revision 2, we removed "significant" from the Abstract and Results in lines 64 and 304, and p-values in lines 70 and 325. Upon review, we revised lines 359-360 in the Discussion that appeared to rely too heavily on the p-values.

University of Zimbabwe
Clinical Trials Research Centre

Administrative Office
15 Phillips Avenue, Belgravia, Harare, Zimbabwe
Tel: +263-24-2704890/2704920, Fax: +263-24-2704897

Research Office
10 Routledge Street, Milton Park, Harare, Zimbabwe
Tel: +263-24-252299/27911374, Fax: +263-24-2704897

KNOWLEDGE - DILIGENCE - INTEGRITY
University of Zimbabwe,
Faculty of Medicine and Health Sciences

Figure A. Sensitivity of odds ratio estimate to varying length-for-age Z-score (LAZ) binary categorization

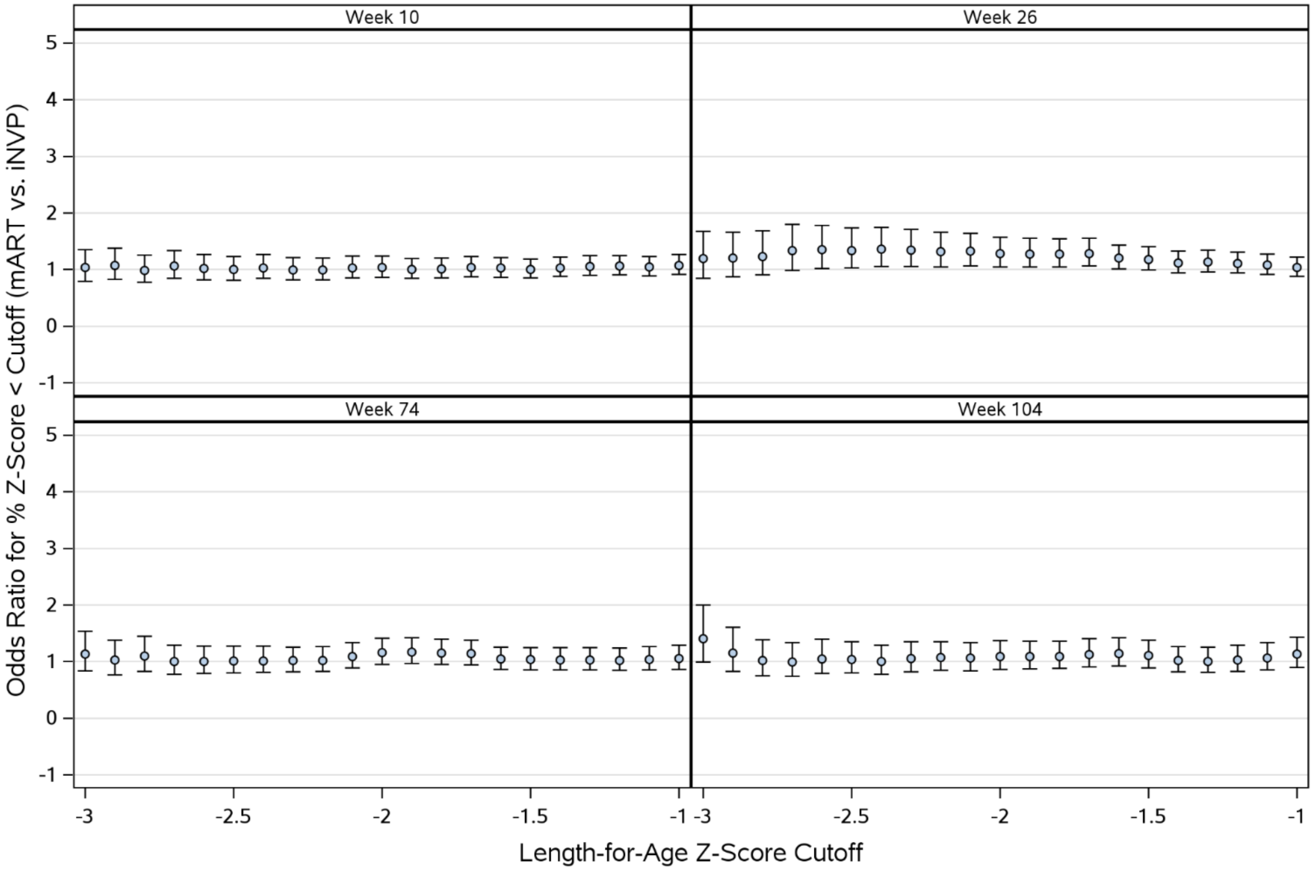

Abbreviations: *iNVP—infant nevirapine prophylaxis; mART—maternal antiretroviral treatment; %—percent*

University of Zimbabwe - Clinical Trials Research Centre
Saving Lives Through Innovative Research Strategies

University of Zimbabwe
Clinical Trials Research Centre

Administrative Office
15 Phillips Avenue, Belgravia, Harare, Zimbabwe
Tel: +263-24-2704890/2704920, Fax: +263-24-2704897

Research Office
10 Routledge Street, Milton Park, Harare, Zimbabwe
Tel: +263-24-252299/27911374, Fax: +263-24-2704897

University of Zimbabwe,
Faculty of Medicine and Health Sciences

Figure B. Sensitivity of odds ratio estimate to varying weight-for-age Z-score (WAZ) binary categorization

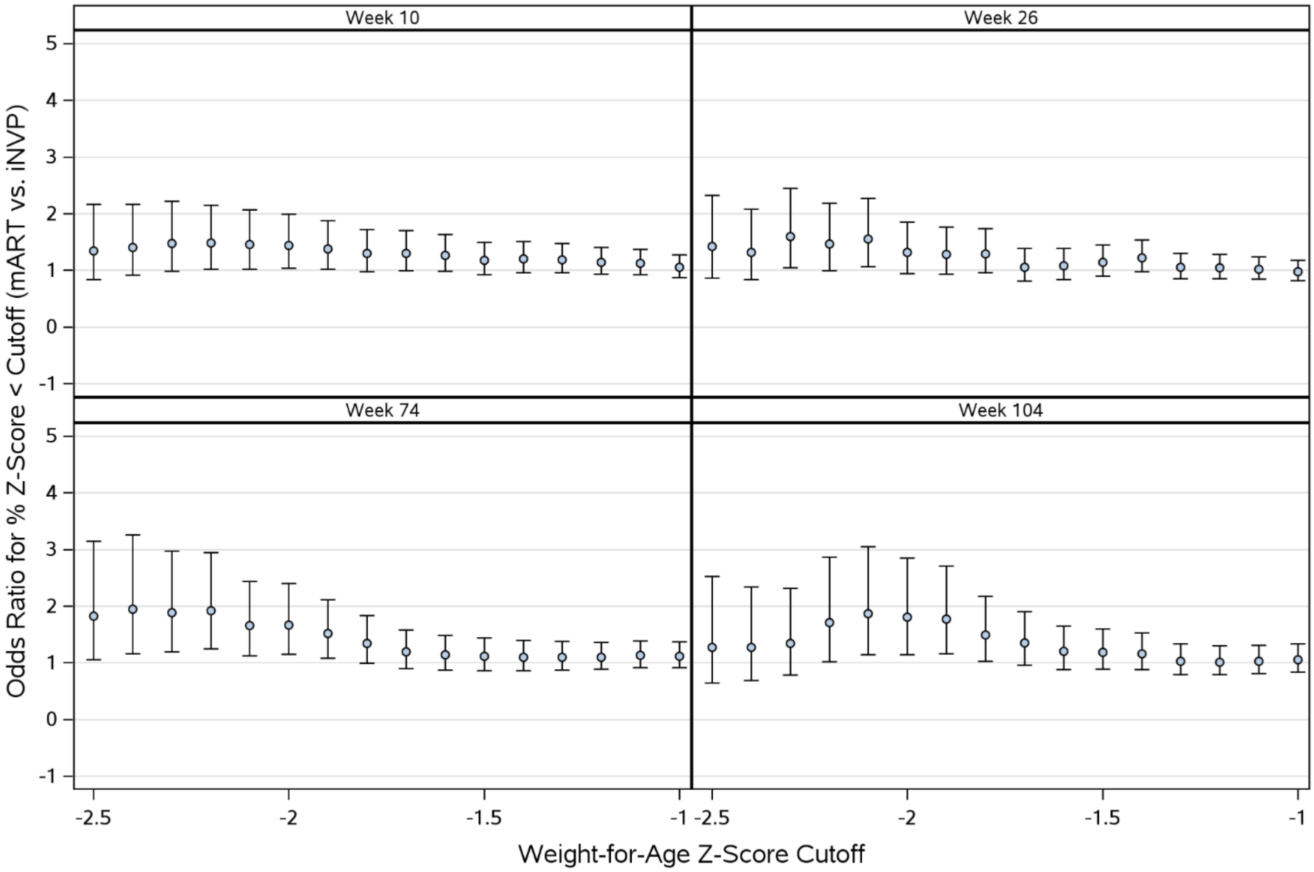

<u>Abbreviations</u>: *iNVP—infant nevirapine prophylaxis; mART—maternal antiretroviral treatment; %—percent*
